# Chilean Supreme Court ruling on the protection of brain activity: neurorights, personal data protection, and neurodata

**DOI:** 10.3389/fpsyg.2024.1330439

**Published:** 2024-02-27

**Authors:** María Isabel Cornejo-Plaza, Roberto Cippitani, Vincenzo Pasquino

**Affiliations:** ^1^Instituto de Investigaciones en Derecho, Universidad Autónoma de Chile, Santiago, Chile; ^2^Department of the Constitutional Law, Universidad Nacional de Educación a Distancia, Madrid, Spain; ^3^Instituto Nacional de Estudios de Derecho Penal, Mexico City, Mexico; ^4^Institute of Applied Physics, Consiglio Nazionale delle Ricerche, Florence, Italy; ^5^Department of Law, Università degli Studi di Perugia, Perugia, Italy

**Keywords:** neurodata, neurorights, GDPR, Supreme Court judgment, artificial intelligence, neuroenhancement, ludic neurotechnology

## Abstract

This paper discusses a landmark ruling by the Chilean Supreme Court of August 9, 2023 dealing with the right to mental privacy, originated with an action for constitutional protection filed on behalf of Guido Girardi Lavin against Emotiv Inc., a North American company based in San Francisco, California that is commercializing the device “Insight.” This wireless device functions as a headset with sensors that collect information about the brain’s electrical activity (i.e., neurodata). The discussion revolves around whether neurodata can be considered personal data and whether they could be classified into a special category. The application of the present legislation on data (the most obsolete, such as the Chilean law, and the most recent EU law) does not seem adequate to protect neurodata. The use of neurodata raises ethical and legal concerns that are not fully addressed by current regulations on personal data protection. Despite not being necessarily considered personal data, neurodata represent the most intimate aspects of human personality and should be protected in light of potential new risks. The unique characteristics of neurodata, including their interpretive nature and potential for revealing thoughts and intentions, pose challenges for regulation. Current data protection laws do not differentiate between different types of data based on their informational content, which is relevant for protecting individual rights. The development of new technologies involving neurodata requires particular attention and careful consideration to prevent possible harm to human dignity. The regulation of neurodata must account for their specific characteristics and the potential risks they pose to privacy, confidentiality, and individual rights. The answer lies in the reconfiguration of human rights known as “neurorights” that goes beyond the protection of personal data.

## 1 Introduction

This text analyzes ruling rol N 1.080–2020 issued on August 9, 2023, by the Supreme Court of Justice of Chile. The ruling arose from an action for the protection of the rights recognized by the Constitution filed on behalf of Guido Girardi Lavin against Emotiv Inc. The company is headquartered in San Francisco, California, and sells portable neuroenhancement products that utilize electroencephalography technology. They also offer neuro-headphones, software development kits, software, and mobile applications to market and sell their Insight device in Chile. This wireless device functions as a headset with sensors that collect information about the brain’s electrical activity (i.e., neurodata).

From a technical standpoint, neurodata typically consist of information regarding the electrical activity of the brain and nervous system. In most cases, this data cannot be used to identify specific individuals, except in highly controlled experimental settings.

According to another definition, neurodata are data derived from the activity of the nervous system of an individual and constitute highly sensitive personal data because they reveal aspects of the internal mental activity of an individual. This internal mental activity is the essence of his personality, so that the protection of this internal forum forms an inseparable unity with the protection of human dignity and, therefore, also with human rights (CJI/OEA Inter-American Juridical Committee, 2023).

On the other hand, neurodata, even if collected and stored in an anonymous manner, as affirmed by the company Emotiv Inc., may be converted into personal data if used in connection with other data.

This data include details of the gestures, movements, preferences, reaction times, and cognitive activity of the device user.

The device is alleged by Mr. Girardi to inadequately protect the user’s brain information privacy, violating constitutional guarantees.

Furthermore, it is noted that neurodata may be susceptible to risks such as reidentification, hacking of brain data, unauthorized reuse, commercialization, digital surveillance, and the collection of brain data for purposes not approved by the individual. On the topic of informed consent, the ruling emphasizes that it is a crucial requirement in scientific research involving human subjects.

One of the points of contention is due to the appellant Guido Girardi Lavin subscribing to an adhesion contract with Emotiv Inc., since if he does not accept the terms of service of Emotiv Inc.’s software, he cannot make optimal use of the device. In addition, the company only releases the information related to the user’s neurodata if the user has subscribed to a paid account called PRO, but by not contracting this additional service, the user’s information is stored in Emotiv Inc.’s cloud, not allowing the user to export or import any record of his neurodata.

Emotiv Inc. highlights that users are provided with detailed terms and conditions and give explicit consent to data processing, which the complainant did to begin using the device.

Emotive Inc. claims to have complied with all the provisions of Law N 19.628 on the Protection of Private Life of Chile, not only with this obsolete standard from 1.999 of particular relevance is Article 11 of Law N 19. 628, which requires due diligence in the care of personal data by those responsible for records or databases of personal data, and Article 13 of the same law, which establishes the right to cancel or block the use of one’s personal data.

A report requested from the health authority confirms that the Insight device is being marketed without all relevant authorizations and has not been evaluated or studied by the health authority. A report was also requested from the National Customs Service, which informs that the Insight device requires a Customs Destination Certificate; this has not been found.

However, Emotiv points out that the neurodata were subjected to pseudonymization treatment to safeguard user data. They claim that personal data are stored only when necessary and that users can always revoke their consent to the processing of brain data. The company highlights that its research data are completely anonymous and treated as statistical data, in accordance with Chilean law.

In addition, the company denies that there are real health risks since the device it markets is for recreational or commercial use and not therapeutic.

In fact, there is a section of scientific doctrine that highlights some contrary considerations, pointing out some possible risks even for what is defined as transcranial Direct Current Stimulation (tDCS) put in place by the user himself (Do-It-Yourself users).

It was shown that stimulation extends well beyond the regions beneath the electrodes and that Current flows between electrodes and can affect the function of various structures along its path (Datta et al., 2009).

Indeed, in a series of studies conducted on the subject, which later resulted in the publication of “An Open Letter Concerning Do-It-Yourself Users of Transcranial Direct Current Stimulation” it was pointed out that Enhancement of some cognitive abilities may come at the cost of others (Dubljevic, 2019). Changes in brain activity (intended or not) may last longer than a user may think, Small differences in tDCS parameters can have a big effectand that tDCS effects are highly variable across different people. As a result, in tDCS the tissue stimulated and effects induced are less deterministic than a user may think, significant tradeoffs may be part of the bargain for functional gains, and whatever brain changes occur may be long-lasting—for better or worse (Wurzman et al., 2016).

This is the first worldwide judgment on “neurodata,” which refers to data obtained using neurotechnologies. Neurotechnology encompasses any electronic method or device that interfaces with the nervous system to monitor or modulate neural activity (Goering et al., 2021).

This paper focuses on the analysis of the Girardi/Emotiv judgment to clarify, through the reasoning of the Chilean judges, if and how neurodata are regulated and protected by the present legislation or if it is necessary to adopt specific legislative instruments.

## 2 Analysis of the judgment from the viewpoint of the protection of personal data

### 2.1 Application of the legislation on protection of personal data

In his appeal, Mr. Girardi claims that the device of Emotiv Inc. uses and stores his brain data, with risks including the following: (i) reidentification, (ii) hacking of brain data, (iii) unauthorized reuse of brain data, (iv) commercialization of brain data, (v) digital surveillance, and (vi) capture of brain data for purposes not consented to by the individual. Such a situation would lead to a violation of Chilean Law N 19.628 on the protection of personal data, in particular Article 11 on due diligence in the care of personal data by the controller. In addition, the appeal alleged noncompliance with Article 13 of the same law on the right of individuals to cancel or block their personal data, since, even when the user account of Emotiv Inc. is closed, the company retains brain information for scientific and historical research purposes.

On its side, the respondent company affirms that the processing of brain data complies with Chilean legislation as well as with the stricter General Data Protection Regulation of the European Union (henceforth “GDPR”).^1^ As a matter of fact, Emotiv Inc. argues that the use of the device is subject to informed and express consent to the processing of personal data, and the individual is allowed to revoke consent to this processing. Such rights are explicitly stated in the privacy policy accompanying the Insight device.

Finally, regarding the data for scientific and historical research, the respondent company points out that the data are completely anonymized, encrypted, and kept securely and separately from the personal data of Insight’s users, so they are data that acquire the legal nature of statistical data following Article 2 letter (e) of Law N 19.628, according to which data that cannot be associated with an identified or identifiable owner are outside the scope of application of the legislation protecting personal data.

### 2.2 Neurodata as personal data

As mentioned above, the controversy refers to “brain data” arising from the use of the device as “personal data.”

This is confirmed by the Organisation for Economic Co-operation and Development (OECD), which argued that data derived from neurotechnologies are personal data because they are “data relating to the functioning or structure of the human brain of an identified or identifiable individual that includes unique information about their physiology, health, or mental states.”^2^

However, due to the characteristics of neurodata and of the neurotechniques, the application of the protection of personal data is not so easy.

As a matter of fact, the aforementioned regulations apply when the data are personal and therefore when they allow the identification of a specific natural person (see Article 2 (f) Law 19,628; Article 4 (1) GDPR).

Considering the broad interpretation of personal data by the Court of Justice of the European Union, any information “about” the individual in question is included.^3^

Additionally, article 4 (1) of the GDPR considers identifiability during processing rather than collection. Thus, information that today would not be associable to specific individuals could become so as a result of technical developments. An analogous case has been the human biological material preserved in collections formed, for medical diagnostic reasons for example, in times when there was no possibility of identifying (except with the association of name and surname) to whom a tissue sample could belong. Today, this identification is possible, and therefore this material contains personal data, but it was collected without observing the present requirements (informed consent among others).

The situation is complicated by the fact that neurodata may have a dynamic content of information (being an evolving technology), in the sense that today it is not easy to distinguish how in the coming years it will be possible to read the same set of data and which will be disaggregated.

### 2.3 Neurodata as special category data

Another question is whether neurodata can be considered “sensitive data” (see Article 2 (g) Law N 19. 628; see also the definition of “special category data” provided for by Article 9, paragraph 1, GDPR). Such data (concerning the physical or moral characteristics of persons, personal habits, racial origin, ideologies and political opinions, religious beliefs or convictions, physical or mental health conditions, and sexual life) cannot be processed, except in cases specified by the law or on the basis of the consent of the data subject (see Article 10 of Law N 19.628; Article 9, paragraph 1, GDPR).

Neurodata are not seen as special categories of data by the abovementioned regulations.

However, they could be considered health-related data when they derive from medical diagnostic activities, or as biometric data.

Biometric data are those obtained from specific technical processing relating to the physical, physiological (e.g., facial recognition, fingerprints, finger geometry, iris recognition, vein recognition, retina scanning), or behavioral characteristics of a physical person (e.g., handwriting patterns or gait) that can be used to form a unique identification of that person (Metzger, 2019).

Neurodata might be also considered sensitive data to the extent that they can reveal “racial or ethnic origin, political opinions, religious or philosophical beliefs, or trade union membership … or data concerning the natural person’s sex life or sexual orientation.”

According to the literature, the use of neurotechnologies can already lead to the identification of the age, sex, and even sexual orientation of a person (Alexander and Sufka, 1993; Carrier et al., 2001). Perhaps other information will also be obtained in the future through the examination of neurodata, such as guilt (Brown and Murphy, 2010) or political leanings (Kanai et al., 2011). Moreover, brain data could be used to read a person’s thoughts and foresee his or her intentions (MacKellar, 2019). However, the latter applications seem more like science fiction scenarios (as in Steven Spielberg’s film *Minority Report*, based on Philip Dick’s novel) or laboratory cases in experimental phases.

Another hypothesis may be that neurodata are a new special category of data. Indeed, neurodata contain a representation of psychic activity, both conscious and subconscious, and correspond to the most intimate aspect of human personality, which is protected by Article 10 of Law N 19. 628 or Article 9 of GDPR against possible forms of discrimination^4^.

The fact that neurodata are not explicitly provided for should not be an obstacle in view of the reason for identifying the category as the protection of a fundamental right of the individual. This is analogous to what has happened in other cases, as in the case of genetic data that were not initially provided for in European personal data protection but that qualified interpreters considered to be sensitive data.

Similarly, the Supreme Court, by interpretation of the legislation into force, could have given neurodata the special protection for sensitive personal data.

As matter of fact, it would be advisable to consider neurodata as sensitive personal data, and, for this reason, they should only be processed if specified by law or by the consent of the data subject. This, because of the potential risks for the person concerned, the purposes of the processing should be identified, and appropriate measures have to be taken in order to not affect the fundamental rights of the person. Such conditions do not seem to be met by Emotiv Inc., which only provides generic information on the future use of data and which affirms the “anonymization” (in reality a pseudo-anonymization) without explaining how this could avoid the reidentification of the data subject.

### 2.4 “Neurodata exceptionalism”

Finally, the regulation of neurodata through Chilean Law N 19.628, the GDPR, or similar legislations may be difficult because neurodata present certain novel characteristics in comparison with other typologies of data.

The legal disciplines on personal data normally do not differentiate data on the basis of their informative content. Also, in the case of sensitive data (or special categories of data), the differences between, for example, genetic data and political opinions are not taken into consideration. However, these differences are relevant in the protection of the rights of the individual. For example, genetic data can provide much information on a specific individual (e.g., health status, physical characteristics, origins) and on other persons forming part of his/her biological family, while political opinion data only refers to a particular opinion.

For this reason, the expression “genetic exceptionalism” (Cippitani, 2018) is used to refer to the necessity of considering the specific characteristics of genetic data with respect to other typologies of data.

Similarly, one could talk about “neurodata exceptionalism” to at least guide the application of data protection regulations and, perhaps in the future, to encourage the adoption of specific regulations.

In particular, neurodata represent a model of the mind of a person that might be used to identify the past and future conduct of the individual. For example, virtual reality allows the collection of a set of data of diverse nature that are provided by the immersive experience and that can be processed by artificial intelligence systems for several possible purposes: marketing, political communication, providing evidence in trial, and so on.

Neurodata have various properties that could be called functional and structural. In this sense, the more transient or cosmetic characteristics might not have the relevance implied by neurodata that possess permanent neural material of an individual (from which permanent structural characteristics can be extracted), while the functional properties may be episodic (in the sense that they respond to stimuli such as cosmetic neuroenhancement). This distinction, although it has not been addressed in data law, could be useful to demonstrate that structural data should have greater rights protection because they could violate the right to privacy to a greater extent, for example, when considering that storage of this data can lead to the detection of more perennial characteristics of the holder of this type of data. Likewise, the right to identity could be altered with neurotechnologies that modify structural neurodata to a greater extent; finally, psychological integrity could also be affected, even irreversibly (Kandel et al., 2021; Wajnerman Paz, 2022; Lavazza and Giorgi, 2023; Ligthart, 2023).

In the face of such scenarios, the current level of protection offered by data protection legislation may be insufficient or inadequate, due to the fact that the current legislation takes into consideration the specific and isolated data and not the information elaborated from such data.

One wonders what Girardi’s defense would have argued if the defendants had argued that the issue of neurodata was not regulated by either the outdated Personal Data Protection Act or the current constitutional law. They would probably have pointed out that the “protection of neural activity” implies a broad principle of interpretation, which certainly subsumes the protection of mental activity, including neurodata and its processing, since there is no specific provision dealing with it. It is important to note that there is currently a bill in the Chamber of Deputies that considers the protection of mental integrity and establishes neurodata as sensitive data.

## 3 The security of neurotechnologies

Another issue to which the judgment of the Supreme Court of Justice of Chile refers is the question of the security of neurotechnologies.^5^ On the other hand, paragraph 6 of the judgment is a reminder that the 1999 UNESCO Declaration on Science and the Use of Scientific Knowledge requests that science and technologies must contribute to the achievement of collective and individual safety and security (see Cippitani, 2023a).

Indeed, the literature shows that neurotechnologies may cause problems in the security of individuals (Zimmerman, 2018). For example, the proposed EU law on artificial intelligence refers to many issues that can be associated with the security of neurotechnologies and neurodata, such as the artificial intelligence systems used by public authorities for law enforcement purposes that use biometric data or polygraphs or that detect the “emotional state”^6^ of a person considered “high risk” (see recital 38 of the law on AI), with the consequent need to apply the specific rules provided for by the law in terms of authorization and control.

In any case, in the judgment at issue, the question of security is not further developed. Instead, the Chilean Supreme Court excludes, for example, that Insight could be authorized by the Instituto de Salud Pública and the Ministry of Health. This is because, according to this authority, on the basis of Article 111 of the Chilean Health Code, it does not require medical authorization or to be registered in the national health registry to be commercialized. Under other legislations, such as EU law, the device should be subject to stricter control. As a matter of fact, according to Regulation (EU) 2017/745 on medical devices, even products considered by the manufacturer to be only cosmetic or for another nonmedical purpose fall under the discipline of that regulation, at least regarding the application of risk management and, if necessary, with regard to clinical safety assessments (see Article 1(2) and Appendix XVI of the regulation).

## 4 Protection of consumers

The necessity of ensuring security in the use of the device also implies the question of the protection of consumers (Cippitani, 2023b, p. 98 ff.). Although the topic of consumer law is not directly discussed in this ruling, a complete analysis must clarify how the marketing of the disputed device impacts the degree of consumer protection.

The first question to be asked is how business practices and marketing strategies can influence consumer choices and whether we can really speak of consumer awareness. Rather than simply verifying the formal expression of consent, it would seem more appropriate to analyze the process of consent formation itself to detect any anomalies at that stage. This process is influenced by actions carried out by companies, both as a sales strategy and as marketing practices.

Marketing offers businesses the opportunity to identify consumers’ needs in advance (Collesei, 1994). Analysis of consumer economic behavior plays a key role in the marketing choices of professionals, enabling them to identify demand and target supply effectively (Tedeschi, 2000). Take as an illustrative case the study that aimed to clarify if and how different black truffle production techniques could, by altering sensory and volatile profiles, impact consumer acceptance of the product (Phong et al., 2022).

It is observed that the power of commercial communication is such that it can even arouse in the consumer a need that, prior to the communication itself, was in fact completely foreign to his/her psychic and volitional sphere (Alpa, 2002). Therefore, legislators intended to regulate the subject to ensure that marketing communications corresponded as closely as possible to the product or service offered. Specifically, the definition of unfair commercial practice was identified.

The Chilean legislature, in Article 3(B) of Law 19.496, recognizes the consumer’s right to truthful information. In Article 17-L, the law imposes penalties on professionals who, in the context of providing financial services or products, mislead the consumer through fraudulent advertising.

With Directive 2005/29/EC, the European legislator, more specifically than the Chilean legislator, distinguishes between misleading commercial practices (Article 6 ff.) and aggressive commercial practices (Article 8 ff.).

Regarding unfair commercial practices, it is appropriate to note that, compared to the Chilean discipline, the European Union has regulated the phenomenon more specifically, and it might be relevant to analyze the concrete case of Emotiv/Girardi in the light of the European legal system, also taking into consideration how the defendant itself has affirmed that the contractual arrangements it has adopted are perfectly compatible with European law. For the study of the concrete case that led to the issuance of the Girardi/Emotiv ruling, it is appropriate to consider that, once the purchase of the device had been made, the consumer, in order to access the platform to view his data—and thus to use the device in a satisfactory manner—was forced to register an account, giving consent to the Terms and Conditions document.

In the appeal, Girardi claims that he used the software with a free license. Consider that acceptance of the contractual terms in fact perfected the defendant company’s license of a worldwide, nonexclusive, fully paid, free, irrevocable, and perpetual license over the recorded data.

The question to be asked is whether this concession by the consumer to the complainant cannot be regarded as a true contractual counter-performance, in exchange for the ability to use the purchased device. If so, it could be argued that the use of the expression “free license” falls within the definition of a misleading commercial practice, since “A commercial practice shall be regarded as misleading if it contains false information and is therefore untruthful or in any way, including in its overall presentation, deceives or is likely to deceive the average consumer, even if the information is factually correct...” (Article 6, Directive 2005/29/EC). To determine whether this is an “onerous” contractual provision, reference can be made to a doctrine that deems the consent to which a product or service is made conditional as nonfree (Thobani, 2016). It seems plausible to say that for there to be no question of necessary consent, the consumer must be able to use the service while refusing to give his data. It will then be necessary to consider the character of the unwaivability of the service (Lo Surdo, 2003) in order to understand whether the user had, in practice, an alternative way of obtaining the contractual service. In the present case, it seems coherent with the legal system to consider as a real contractual performance the transfer of a license on the data recorded by the device. “In other words, the link of correspondence is considered to exist (for the purpose of applying consumer protections) whenever the consent of the data subject is necessary to process the data, regardless of his active cooperation and actual awareness of the processing” (Thobani, 2021). It seems difficult to consider as “free” the access—obligatory—to the platform, with a consequent, probable implementation by Emotiv of a misleading commercial and aggressive practice (see for example the definition provided by Article 8 of Directive 2005/95/EC).

The judgment at issue provides an opportunity to reflect on the possibility of a paradigm shift regarding aggressive commercial practices and their nature. It should also be considered that it is the business practices themselves that can impact the user’s decision-making process. Thus, there will be, on the one hand, a study aimed at obtaining information about the habits of the potential consumer. On the other hand, it is possible to study how to influence users to make them become consumers.

The neurodata obtained using Insight, which, from what is stated in the company’s own Terms of Use, can be distributed to third parties for “research and experiments,” constitute a valuable set of information in this regard. Once obtained, they could be cross-referenced with other data streams to create a consumer target and profile a certain category of users.

A 2019 study examined three different online profiling models and highlighted their effectiveness. It was observed how using data provided by third parties increases the effectiveness in identifying the audience by up to 123 percent (Neumann et al., 2019). The advent of neurodata transfer for the purpose of targeting, however, has the potential to greatly enhance these activities, enabling companies to conduct increasingly targeted and effective business practices.

In this case, one would have to question the lawfulness of such a practice. The doubt, in fact, is that the use of neurodata for these purposes constitutes undue conditioning, limiting the consumer’s freedom of choice and thus configuring itself as an aggressive commercial practice.

There is a growing need for the introduction of specific regulations regarding this type of data and a rethinking of the mechanisms that protect the consumer from unfair commercial practices, with particular attention given to the use of new technologies that are increasingly capable of influencing the individual’s freedom of choice, inducing him or her more and more to consume.

## 5 Protection of neurodata as a protection of human rights

As mentioned in the previous paragraphs, the judgment of the Supreme Court of Justice of Chile, the first known in the world regarding the use of neurodata, poses several ethical and legal questions that are not solved by the present legislation in Chile or in other countries (e.g., in the European Union).

In addition to the issues concerning the data, security, and protection of consumers, the Girardi/Emotive Inc. judgment emphasizes that neurotechnologies and neurodata are (or should be) disciplined by different rules that regulate multiple aspects of a phenomenon that is complex and dynamic, as happens in other fields subject to the evolution of techno-science (see, for example, the case of technologies using genetic data and human biological materials, Cippitani et al., 2023; Colcelli et al., 2023).

The judgment of the Supreme Court of Justice of Chile poses further important questions.

As underlined by the Girardi /Emotive Inc. judgment, “the development of new technologies involves more and more aspects of the human person—aspects that were unthinkable a few years ago.” The State is expected to act “in order to prevent and anticipate their possible effects, in addition to directly protecting human integrity in its totality, including privacy and confidentiality” (see paragraph 8 of the judgment). Therefore, the judgment points out that privacy is an (important) aspect of human integrity, or, in other words, of dignity and other human rights as cognitive liberty, freedom of thought and identity (Andorno, 2023; Farahany, 2023; Lavazza and Giorgi, 2023; Ligtharts et al., 2023).

It is particularly relevant that in the seventh section of the verdict, the Supreme Court of Justice of Chile emphasizes the importance of obtaining informed and specific consent from individuals involved in scientific activities, citing both international sources and national legislation on scientific research (Law 20.120).

Therefore, the respondent’s explanation that the data collected from Insight users becomes anonymous and thus transforms into publicly available statistical information omits the requirement for explicit consent for research purposes. Furthermore, this allows the ruling out of the possibility of such consent being deemed implicitly given through other consents or approvals provided by the purchaser of a certain device as a client or consumer. Specific consent indicating the purpose and aim of the corresponding research is required.

The abovementioned judgment of the Supreme Court of Justice of Chile refers to Law N 21.383, which modifies the Political Constitution of the Republic to establish scientific and technological development in the service of the people. The final clause of article 19, number 1, now specifies the following:

“Scientific and technological development will be in service to individuals and carried out with respect for life and physical and psychological integrity.

The law will regulate the requirements, conditions, and limitations for its use in individuals, with particular emphasis on safeguarding brain activity and the information derived from it.”

The tenor of the phrase to protect “brain activity, as well as the information coming from it...” “has raised problems of interpretation of the law and ethical concerns (Borbón and Borbón, 2021; Zúñiga-Fajuri et al., 2021; Bublitz, 2022; Fins, 2022; Rommelfanger et al., 2022; Cornejo-Plaza, 2023b).

One of these has been the discussion of the reductionism of locating the protection of the person in an organ such as the brain. But it has been suggested that a broad interpretation should be considered, integrating the psychic and mental aspects of the person (Cornejo-Plaza, 2021; Wajnerman Paz, 2022; Andorno, 2023).

On the other hand, article 19, paragraph 4, which predates the neurorights reform, also provides for the protection of both private life and personal data. This norm could be interpreted in contradiction with the reform, since if this clause protects the private life of the person and his or her data, ergo mental life means falling back into Cartesian dualism, having a split vision of the mind and the body. Moreover, it would be understood that neurodata are not included in the category of personal data protection.

## 6 The “neurorights” within Chilean Law

In the frame of the protection of “human integrity,” the abovementioned paragraph 8 of the judgment emphasizes the necessity of “the neurorights at stake, such as mental privacy and cognitive freedom.”

Neurodata can affect mental integrity because, among other things, it can “control mental states, decode them by providing behavioral information...” (Zohny et al., 2023). For its part (Ligtharts et al., 2023) establishes the possibility that “thoughts and feelings can be observed, not only indirectly through behavior, but also through multimodal data analysis, in which data on brain states play an important role, it can have harmful consequences in the individual, his social and political interactions” (Zohny et al., 2023).

“Decoding brain data may 1 day reveal mental information,” so neurodata affects not only mental privacy but also mental integrity. If it is considered that the facts denounced have violated both psychological integrity and privacy (whereas 9), the Supreme Court should have based its decision on the decision does not refer to this point, thereby losing the interpretive opportunity to establish jurisprudence on the real basis of neurodata protection.

In fact, as the legal literature argues, in the field of neurotechnologies it is necessary to consider the proposed reconfiguration of human rights known as “neurorights”—such as cognitive freedom or mental privacy, psychological integrity, decisions free from algorithmic bias, and equity in access to cognitive augmentation technologies—as a natural evolution of human rights in digital environments but with obvious emphasis on the impact of neurotechnologies (Bublitz, 2020; Andorno, 2023; Cornejo-Plaza and Guiñazú, 2023). These are issues that go beyond the protection of personal data (see Cornejo-Plaza, 2021; Cornejo-Plaza and Saracini, 2023).

The judgment mixes terminologies such as privacy and psychological integrity (Hildt, 2022; Andorno, 2023; CJI/OEA Inter-American Juridical Committee, 2023; Lavazza and Giorgi, 2023; Ligthart 2023), which implies use of the theoretical approach of neurorights.

However, further dogmatic elaboration of these new configurations is needed, as they are not equivalent when addressing the problem of governance of neurotechnologies and brain data.

Another noteworthy point is that the ruling does not address neurotechnological devices that are able to neurophysiologically identify an individual; nor does it refer to how their dissemination or transaction would affect individuals’ fundamental rights, including neurorights.

In any case, this ruling marks a groundbreaking moment and exhibits a distinct commitment to safeguarding and recognizing a novel approach to shaping certain emerging human rights in the face of rapid advancements in AI and related technologies (Yuste et al., 2021).

The legal recognition of neurorights has been realized by Chilean legislation, making Chile a pioneer country in this field (Mantegna, 2023). Along the same lines, Mccay maintains that: “In addition to its broader importance for neurotechnology and human rights, the change could be considered a milestone in the protection of neurodata (data derived from the brain or nervous system). This legislative step has set a precedent and other countries, including Brazil and Mexico, are now also considering constitutional change, and the US-based Neurorights Foundation has been active in related advocacy” Mccay (2023).

The bill emphasizes the significance of safeguarding neurodata and mental integrity, according to Bulletin 13.828–19. Its aim is to oversee the progression and advancement of neurotechnologies while preserving neuronal rights.

The legislation’s goal is to apply protective measures for brain activity and to recognize the importance of neurorights. It is suggested that data obtained through specific neurotechnologies may infringe on fundamental rights, such as privacy, mental integrity, cognitive liberty, freedom of thought and identity (Ienca and Andorno, 2017; Yuste et al., 2017; Lavazza, 2018; Wajnerman Paz, 2021; Farahany, 2023; Ligtharts et al., 2023; Muñoz, 2023).

The bill on protecting neurorights provides regulations for recreational neurotechnologies, establishing informed consent and adequate information as restrictions on inappropriate use by users (Cornejo-Plaza, 2023a; Ligtharts, 2023; Zohny et al., 2023). This solution is appropriate if one considers the potential risks of nontherapeutic applications of neurotechnologies to individuals’ health. Currently, this bill is in its second constitutional process in the House of Representatives.

According to Chilean legislation, the Supreme Court of Justice’s ruling explicitly cites Bulletin 13.828–19, noting that neurodata is sensitive and biometric data and therefore falls under the Data Protection Act 19.698. This type of data is not always exposed in therapeutic and biomedical relationships but extends beyond them. The ruling refers specifically to the collection of neurodata in the commercial and/or recreational digital world. In this context, using the data without the user’s consent could pose an unknown risk that can only be speculated upon (Cornejo-Plaza, 2023a).

Neurodata can be considered sensitive, personal, and biometric data, contrary to what is stated in the European regulation; being so means it is not possible for Emotiv Inc. to appropriate such personal data, although it cannot identify the user for now—as the sentence itself says, it is necessary to observe the development of technology, which suggests that in a while the identification of the user may be possible through neurodata, whether or not they are anonymized. Thus, the issue of consent is a false safeguard put forward by the company, since even if the user gives his consent, and thus his data can be considered personal, his exposure also makes the owner of such data potentially vulnerable.

## 7 Discussion and conclusion

The adequacy of the neurorights regulation cited in the judgment passed by the Chilean Supreme Court, which establishes protection for brain data, is under debate as regards the use of devices for nontherapeutic commercial purposes that collect neurodata with the user’s consent.

The article examines the Chilean ruling on neurodata and its regulatory application in the present legislation. It raises the question of whether neurodata should be classified as personal data and whether they require special categorization. Furthermore, it explores the significance of informed consent and the safeguarding of neurodata within the commercial sphere.

The need to consider the consumer’s perspective and protect them from manipulative commercial practices that may influence their informed consent is mentioned.

The proposed reconfiguration of human rights, called “neurorights,” extends beyond safeguarding personal data and security and tackles concerns related to mental integrity, mental privacy, cognitive liberty, freedom of thought, algorithmic biases, and an equitable approach to cognitive augmentation technologies.

This article concludes that the regulation of neurorights must address the ethical and legal challenges that arise from the use of neurodata and disruptive neurotechnologies. Additionally, it stresses the need to research and discuss neurorights continually, enabling laws to keep up with technological advancements and societal requirements.

The highlighted need for regulation aims to specifically address the unique characteristics of neurodata and the potential risks they pose to individuals’ privacy, confidentiality, and others fundamental rights.

It is necessary to develop legal frameworks that protect the privacy, confidentiality, and rights of individuals.

We could point out that neurodata have diverse functional and structural properties, so to speak, in the sense that the structural characteristics of neurodata may be permanent, while the functional properties may be episodic in the sense that they respond to stimuli such as cosmetic neuroenhancement. Although this distinction has not been addressed in the data law. Likewise, the right to identity could be altered with neurotechnologies that modify structural neurodata to a greater extent. Finally, psychological and mental integrity could also be affected to a greater extent, so we consider that a classification of neurodata of this nature makes it clear that neurorights are more appropriate to response than data law to safeguard fundamental rights against the disruptive use of neurotechnologies, whether therapeutic or recreational.

## Author contributions

MC-P: Conceptualization, Formal analysis, Investigation, Methodology, Supervision, Validation, Writing – original draft, Writing – review & editing. RC: Conceptualization, Formal analysis, Funding acquisition, Investigation, Methodology, Resources, Validation, Writing – original draft. VP: Conceptualization, Formal analysis, Methodology, Writing – original draft, Writing – review & editing.

## Footnotes

^1^Regulation (EU) 2016/679 of the European Parliament and of the Council of April 27, 2016, on the protection of natural persons with regard to the processing of personal data and on the free movement of such data, and repealing Directive 95/46/EC (General Data Protection Regulation).

^2^OECD, Recommendation of the Council on Responsible Innovation in Neurotechnology (2020), https://legalinstruments.oecd.org/en/instruments/OECD-LEGAL-0457.

^3^Court of Justice, judg. December 20, 2017, C-434/16, Peter Nowak v. Data Protection Commissioner, ECLI:EU:C:2017:994, paragraph 34.

^4^See Article 29 Data Protection Working Party in its document *Working Document on Genetic Data*, adopted on March 17, 2004 (paragraph III, p. 5).

^5^See “The risks and challenges of neurotechnologies for human rights.” https://unesdoc.unesco.org/ark:/48223/pf0000384185.

^6^The Artificial Intelligence ACT refers to the “emotion recognition system,” which means an AI system for the purpose of identifying or inferring emotions or intentions of natural persons on the basis of their biometric data (see Article 3 (34)).
